# Gender Differences in Vogt-Koyanagi-Harada Disease and Sympathetic Ophthalmia

**DOI:** 10.1155/2014/157803

**Published:** 2014-03-05

**Authors:** Yujuan Wang, Chi-Chao Chan

**Affiliations:** ^1^Immunopathology Section, Laboratory of Immunology, National Eye Institute, National Institutes of Health, 10 Center Drive, 10/10N103, Bethesda, MD 20892-1857, USA; ^2^Zhongshan Ophthalmic Center, Sun Yat-sen University, Guangzhou 510060, China

## Abstract

Vogt-Koyanagi-Harada disease (VKH) and sympathetic ophthalmia (SO) are types of T-cell mediated autoimmune granulomatous uveitis. Although the two diseases share common clinical features, they have certain differences in gender predilections. VKH classically has been reported as more prevalent in females than males, yet some studies in Japan and China have not found differences in gender prevalence. Male patients have a higher risk of chorioretinal degeneration, vitiligo, and worse prognosis. Conversely, the changing levels of estrogen/progesterone during pregnancy and the menstrual cycle as well as higher levels of TGF-*β* show a protective role in females. Potential causes of female predilection for VKH are associated with HLA-DR and HLA-DQ alleles. SO, a bilateral granulomatous uveitis, occurs in the context of one eye after a penetrating injury due to trauma or surgery. In contrast to the female dominance in VKH, males are more frequently affected by SO due to a higher incidence of ocular injury, especially during wartime. However, no gender predilection of SO has been reported in postsurgical cases. No clinically different manifestations are revealed between males and females in SO secondary to either ocular trauma or surgery. The potential causes of the gender difference may provide hints on future treatment and disease evaluation.

## 1. Introduction

Vogt-Koyanagi-Harada disease (VKH) and sympathetic ophthalmia (SO) are both considered ocular T-cell mediated autoimmune diseases. Although the pathogenesis and etiologies are different, the two entities share common clinical manifestations as well as similar pathological and immunohistochemical features [1–3]. Moreover, prompt and thorough treatment is essential for both VKH and SO. Systemic presentations and clinical history are important features that differentiate VKH from SO. Extraocular findings such as dysacusis, vitiligo, poliosis often develop in VKH but are rare in SO. Since females are more susceptible to a variety of autoimmune diseases, such as systemic lupus erythematosus, multiple sclerosis, and rheumatoid arthritis [4–6], this paper reviews the literature of VKH and SO focusing on whether gender predilection exists in these two diseases.

### 1.1. Clinical Aspects of VKH

VKH disease is a multisystemic disorder that involves the eyes, ears, skin, hair, and meninges. Ocular manifestations are characterized by bilateral granulomatous panuveitis with exudative retinal detachments. In the majority of VKH patients, the second eye becomes involved within 2 weeks after initial presentation. Overall, females are more frequently affected with VKH than males [7–9], although several studies found no such gender predilection [10, 11]. VKH tends to affect patients from Asian, Middle Eastern, Hispanic, and Native American populations [7, 12].

The typical progression of VKH includes 4 stages: the prodromal, uveitic, chronic, and chronic recurrent stages. The prodromal stage occurs 3–5 days before the ocular disease, mimicking a systemic viral-like presentation. The uveitic stage, which may last for several weeks to months, is characterized by acute anterior uveitis with mutton-fat keratic precipitates, aqueous cells and flare, iris nodules, and synechiae. Severe changes in the posterior segment include vitritis, optic disc swelling, retinal edema, hemorrhages, nonrhegmatogenous exudative retinal detachment, subretinal fibrosis, disciform scars, and RPE abnormality. The focal yellowish-white nodular lesions, known as Dalen-Fuchs nodules, are presented at the subretinal pigment epithelium (RPE) level in the peripheral retina. The Hallmark findings in the uveitic stage are multifocal detachments of the neurosensory retina. Depigmentation of the perilimbus (Sugiura's sign) and a pale fundus (sunset-glow fundus) are revealed in the chronic stage. The chronic recurrent stage often presents other complications such as cataract, glaucoma, subretinal neovascularization, and subretinal fibrosis [13, 14]. Recurrence mainly involves the anterior segment. Extraocular presentations including vitiligo, poliosis, alopecia, and dysacusis may also develop during the chronic or chronic recurrent stages.

### 1.2. Clinical Aspects of SO

SO is a rare bilateral granulomatous uveitis that occurs after the uvea of one eye is subjected to a penetrating injury due to trauma or surgery. The injury to one eye (known as the* exciting eye*) also results in an inflammatory response in the noninjured, contralateral eye (known as the* sympathizing eye*). Unlike the multisystemic involvements of VKH, extraocular manifestations are rare in SO. Due to the rarity of disease and great improvements in modern surgical techniques, the disease rarely occurs; thus, it is difficult to estimate the prevalence of SO. An earlier study published the incidence of SO after a penetrating injury to be approximately 2% prior to 1950 [15]; lower incidences—0.2–0.5% following ocular trauma and 0.01% following ocular surgery—have been reported in more recent studies [16, 17]. The interval between ocular injury and the SO onset varies to a large extent, ranging from 5 days to 66 years [16, 18, 19]. In general, 65% of SO occurs between 2 weeks and 2 months after injury and 90% occurs within 1 year [16, 20].

The clinical presentations are identical in both trauma- and injury-induced SO with an insidious onset. The classic presentation of SO is characterized by an acute granulomatous inflammation in the anterior segment with mutton-fat keratic precipitates, aqueous cells and flare, iridocyclitis, and posterior synechiae. Moderate to severe vitritis with choroidal thickening and infiltration as well as optic disc swelling generally occurs in the posterior segment [21–24]. The presence of Dalen-Fuchs nodules, measuring 60 to 700 *μ*m in diameter, is typical and is most often found at the midperipheral retina in SO [25]. In extremely rare situations, patients with SO may experience extraocular symptoms, such as hearing loss, headache, vitiligo, and alopecia [26, 27].

## 2. Gender and VKH

### 2.1. Gender Differences in Prevalence and Incidence

Most studies have reported that females are affected with VKH more frequently than males (Table 1). Earlier studies in North America have noted that 60–78% of VKH patients are females [10, 12, 13, 28, 29]. By reviewing 75 VKH patients seen at the National Eye Institute between 1978 and 1996, 78.7% of patients were females [7]. In other areas, the recently reported proportions of affected females with VKH are 78.9% in Mexico [9]; 62.9–75% in Saudi Arabia [8, 30, 31]; 65.3% in Tunisia, North Africa [32]; 73.7% in Japan [33]; 84.44% in South India [34]; 70% in Brazil [35]; and 71.1% in Turkey [36]. Sukavatcharin et al. reported that females constituted 62.5% of patients in 48 case series in a Hispanic population [37].

However, some other studies did not show similar findings. Sasamoto et al. found that only 38% of patients were females in their 47 case series of VKH in a Japanese population [38]. Chen et al. and Hou et al. even reported more male patients in more than 500 Chinese with VKH [11, 39]. However, no gender predisposition of the disease was also reported from the same group in other studies [40–45]. Other VKH studies have also reported no gender differences in prevalence; interestingly, most of these conclusions come from studies of Asian populations [46–49]. These data might indicate that there are geographic variations in the gender predilection in the VKH patients [13].

### 2.2. Gender Differences in Clinical Manifestation after the Prevalence/Incidence

Interestingly, ocular manifestations of VKH are variable and race dependent, and the “sunset-glow” appearance is more commonly seen in Hispanic and Asian patients [13]. A study of 134 eyes (67 VKH patients) conducted in Singapore reported that male VKH patients (50 eyes) were clinically associated with a higher risk of chorioretinal degeneration and vitiligo [47]. No other clinical differences have been reported in VKH between male and female patients.

### 2.3. Gender Differences in Prognosis

Several factors have been related to a better prognosis in female patients with VKH. Pregnancy is reported to play a role in VKH prognosis and has a beneficial effect on disease activity [10, 50–52]. In general, two patterns of prognosis during pregnancy have been described [10, 50–52]. In some females with VKH, the ophthalmic presentations improved during pregnancy, but with recurrence after delivery [10, 51, 53]. Several studies have documented less uveitis reactivity with lower numbers/rates of flare-up during pregnancy, but many of these females experienced a rebound in activity within 6 months of delivery [53–55]. However, in the other cases, VKH developed in females during pregnancy who were then cured with corticosteroids without recurrence following delivery [50]. Because VKH is a T-cell mediated autoimmune disease, changes in immunity and humoral constituents during pregnancy may account for the remissions in female patients [51]. In addition, Elias et al. showed that better final visual acuity is positively correlated with female patients, whereas male gender in VKH is significantly associated with worse visual acuity [56].

### 2.4. Possible Explanations for Gender Differences Found in VKH

#### 2.4.1. Relationship with Hormone Changes

Sex hormones, including estrogen and progesterone, are believed to mediate the immune response and account for gender differences in the prevalence of autoimmune diseases [57]. An increased incidence or severity of inflammation has been reported in the late phase of the menstrual cycle for women with asthma, rheumatoid arthritis, and psoriatic arthritis [58–61]. As a systemic autoimmune disorder and subtype of uveitis, VKH is also influenced by hormonal factors [61, 62].

Literature showing VKH amelioration during pregnancy has suggested that sex hormones may influence the course of VKH [10, 51]. To further evaluate the protective role of pregnancy, we used the experimental autoimmune uveitis (EAU) mouse model mimicking human uveitis [63]. Our results suggest that protection from EAU during pregnancy is primarily due to a selective reduction of antigen-specific Th1 responses with only marginal enhancement of Th2 function. These effects may in part be secondary to elevated systemic levels of TGF-*β*. We measured serum levels of the female hormones (estrogen, progesterone, and prolactin), Th1 (IL-2 and IFN-*γ*), Th2 (IL-4, IL-5, IL-6, and IL-10), and TGF-*β* cytokine levels in 4 women with uveitis during their 5 normal, full term pregnancies. Uveitic activities decreased after the first trimester but flared up in early postpartum period, suggesting an association of female hormones and elevated TGF-*β* with uveitis [64].

In addition, Sanghvi et al. evaluated 76 regularly menstruating women with acute anterior uveitis and found that the disease commences more frequently in the postovulatory phase of the menstrual cycle [61]. They concluded that the onset of the acute anterior uveitis is partially dependent on the levels of estrogen and/or progesterone. The withdrawal of these hormones, with their proven anti-inflammatory effects, may provoke the onset of uveitis.

Based on the association of pregnancy and menstrual cycle with VKH, it is important to assess the menstrual history and to consider adjustments of immunosuppressants, such as corticosteroid treatment, during pregnancy and postpartum [51].

#### 2.4.2. Relationship with HLA-DR Genes

Although the precise mechanism of VKH is still not clear, genetic factors are thought to play an important role in VKH [61, 62]. The associations of HLA-DR53, HLA-DR4, and HLA-DQ4 antigens with VKH have been reported [65–68]. Additionally, a strong association with HLA-DRB1∗04:05 allele has been documented in VKH [69, 70]. Recently, Aláez et al. in Mexico compared 76 VKH patients (78.9% females) and 256 healthy controls using the HLA-DQB1/DRB1 genotyping method [9]. They found that HLA-DRB1∗04:05, HLA-DRB1∗04:04, and HLA-DQB1∗03:02 alleles were restricted only to female gender. This study implies a significant association with female gender and HLA in VKH.

## 3. Gender and SO 

### 3.1. Gender Differences in Prevalence/Incidence and Possible Explanation

In trauma-induced SO, males are reported to have a higher prevalence than females [71]; this gender predominance may be attributable to a higher incidence of ocular injury in males, especially during historical wartime [72]. Galor et al. reviewed 85 patients with SO and showed a slight male predilection of male gender (60%) with a higher incidence of traumatic etiology (62%) [73]. We reviewed 32 patients with SO presented at the National Eye Institute, including 23 patients with SO resulting from trauma and 9 resulting from surgery. The numbers of males and females are equally distributed in the case series from 1982 to 1992 [74]. Al-Halafi et al. reported no gender difference in disease incidence in 34 males and 26 females with SO due to ocular injury during a 10-year period in Saudi Arabia [8].  Table 2 summarizes the demographics of SO patients resulting from trauma and surgery. Interestingly, only one report shows significant male predisposition of 30 SO patients [71]. Overall, no gender predilection of SO has been reported in postsurgical cases. This is due to the fact that intraocular surgeries including glaucoma surgery, cataract extraction, and pars plana vitrectomy are equally performed in both male and female patients [20, 74].

### 3.2. Gender Differences in Clinical Manifestation after the Prevalence/Incidence

There are no clinical differences between males and females in SO due to either trauma or ocular surgery.

### 3.3. Gender Differences in Prognosis

Due to the rarity of SO, it is difficult to compare gender differences in the prognosis of SO. However, because SO and VKH share many clinical and pathological similarities, the role of sex hormone and pregnancy could also affect disease severity and presentation, Further clinical and/or experimental studies are required to draw a conclusion.

## 4. Conclusions

Both VKH and SO are types of bilateral granulomatous panuveitis that can lead to severe visual loss without effective management. In addition to clinical features, gender predilection in VKH and SO could provide more appropriate therapies for patients. In VKH, with the protective role of estrogen/progesterone, female patients are better protected and have better prognoses. Moreover, the evidence that certain HLA-DR alleles are exclusively associated with VKH in females implies an important genetic background in the pathogenesis of VKH. In SO, although gender differences only exist in the incidence of ocular trauma, we cannot rule out the possible role of gender-based factors in the initiation, progression, and prognosis of SO. Additional gender-based studies may identify other genes or risk factors related to these two autoimmune diseases.

## Figures and Tables

**Table 1:** Demographic differences of Vogt-Koyanagi-Harada disease (VKH) in the literature.

Author/year	Total: male (%)/female (%); *P* value	Age: mean ± SD (range, years)	Race or region	Reference
Ohno et al./1977	51: 23 (45.1)/28 (54.9) *P* = 0.4852	NA	African American: 13.7% Oriental: 29.4% American Indian: 7.8% Spanish American: 15.7% White: 29.4% Others: 2 (3.9%)	[12]

Snyder and Tessler/1980	20: 8 (40)/12 (60) *P* = 0.3727	39.7 (10–56)	African American: 11 (55%) Hispanic: 7 (35%) White: 2 (10%)	[10]

Belfort Jr. et al./1988	33: 10 (30)/23 (70) *P* = 0.0174**	NA	White: 60% Darkly pigmented: 24% Sansei: 9% African American: 6%	[35]

Sasamoto et al./1990	47: 29 (61.7)/18 (38.3) *P* = 0.1057	41.1 (14–64)	Japan	[38]

Beniz et al./1991	48: 15 (31.2)/33 (68.8) *P* = 0.0072**	33.4 ± 14.5 (15–78)	Hispanic: 75% White: 10.4% African American: 4.2% Oriental: 10.4%	[28]

Rubsamen and Gass/1991	22: 5 (22.7)/17 (77.3) *P* = 0.0060**	35 (13–73)	Hispanic: 54% African American: 23% White: 14% Oriental: 9%	[29]

Moorthy et al./1995	65: 17 (26.2)/48 (73.8) *P* = 0.0000**	32 (7–71)	Hispanic: 51 (78%) Asian: 6 (10%) African American: 4 (6%) Native American: 1 (1.5%) White: 2 (3%) Asian Indian: 1 (1.5%)	[13]

Lertsumitkul et al./1999	75: 16 (21.3)/59 (78.7) *P* = 0.0000**	32.8 ± 12.6 (11–72)	White/native American: 22.7% African/native: American: 52.0% Hispanic: 12.0% Oriental: 12.0% Asian Indian: 1.3%	[7]

Sheu et al./2003	39: 21 (53.8)/18 (46.2) *P* = 0.6368	39.82 ± 12.38	Taiwan	[75]

Wakabayashi et al./2003	19: 5 (26.3)/14 (73.7) *P* = 0.0306**	NA	Japanese	[33]

Sheu et al./2004	31: 19 (61.3)/12 (38.7) *P* = 0.2063	38.6 ± 10.6 (20–63)	Taiwan Chinese	[76]

Tesavibul and Sansanayuth/2005	33: 12 (36.4)/21 (63.6) *P* = 0.1142	35 ± 13.4 (17–67)	Thai	[48]

Sukavatcharin et al./2007	48: 18 (37.5)/30 (62.5) *P* = 0.0801	35 ± 13	Hispanic	[37]

Chee et al./2007	89: 38 (42.1)/51 (57.9) *P* = 0.1347	41.8 ± 14.7 (SE)	Chinese: 75.28% Malays: 14.61% Indians: 5.62% Others: 4.49%;	[46]

Khairallah et al./2007	49: 17 (34.7)/32 (65.3) *P* = 0.0291**	35 (16–54)	North Africa	[32]

Murthy et al./2007	45: 7 (15.6)/38 (84.4) *P* = 0.0000**	37 ± 14.2 (14–63)	South India	[34]

Tugal-Tutkun et al./2007	45: 13 (28.9)/32 (71.1) *P* = 0.0032**	31 ± 14.3 (4–65)	Turkish	[36]

Kiyomoto et al./2007	68: 29 (42.6)/39 (57.4) *P* = 0.2215	43.1 ± 14.2 (16–71)	Japanese	[77]

Al-Kharashi et al./2007	68: 17 (25)/51 (75) *P* = 0.0000**	25 ± 10.3 (7–55)	Saudi Arabia	[31]

Hou et al./2008*	231: 128 (55.4)/103 (44.6) *P* = 0.1001	33.6 ± 12.4	Chinese	[40]

Chee et al./2009	67: 27 (40.3)/40 (59.7) *P* = 0.1103	42.3 (5.4–70.9)	Chinese: 79.1% Others: 20.9%	[47]

Lai et al./2009	35: 18 (51.4)/17 (48.6) *P* = 0.8694	42.5 ± 18.4 (10–72)	Hong Kong Chinese	[78]

Iqniebi et al./2009	30: 12 (40)/18 (60) *P* = 0.2727	NA	Saudi Arabia	[69]

Meng et al./2009*	247: 138 (55.9)/109 (44.1) *P* = 0.0630	33.6	Chinese	[41]

Hou et al./2009*	307: 171 (55.7)/136 (44.3) *P* = 0.0453**	34.3 ± 10.3	Chinese	[39]

Hu et al./2010*	379: 197 (51.9)/182 (48.1) *P* = 0.4596	32.8 ± 9.8	Chinese	[43]

Chee et al./2010	28: 13 (46.4)/15 (53.6) *P* = 0.7055	42.2 (median) (16–77)	Chinese: 64.3% Malays: 21.5% Indians: 7.1% Others 7.1%	[79]

Jiang et al./2010*	382: 210 (55)/172 (45) *P* = 0.0502	33.6 ± 12.4	Chinese	[42]

Shu et al./2010*	385: 201 (52.2)/184 (47.8) *P* = 0.3880	34.1 ± 9.6	Chinese	[44]

Al$\overset{´}{\text{a}}$ez et al./2011	76: 16 (21.1)/60 (78.9) *P* = 0.0000**	42.1 (11–76)	Mexican Mestizos	[9]

Al-Halafi et al./2011	256: 95 (37.1)/161 (62.9) *P* = 0.0000**	29 ± 13	Saudi Arabia	[8]

Chen et al./2012*	519: 290 (55.9)/229 (44.1) *P* = 0.0070**	30.0 ± 13.5	Chinese	[11]

Yang et al./2012	38: 17 (44.7)/21 (55.3) *P* = 0.5152	50 ± 8.4	Hong Kong Chinese	[80]

Chen et al./2012*	451: 243 (53.9)/208 (46.1) *P* = 0.0973	33.8 ± 9.3	Chinese	[45]

Morita et al./2013	85: 37 (43.5)/48 (56.5) *P* = 0.2301	47.1 ± 14	Japanese	[81]

Alam et al./2013	9: 4 (44.4)/5 (55.6) *P* = 0.7440	28 (16–43)	Pakistanis:	[49]

*Study from the same VKH research group in China; **P < 0.05.

**Table 2:** Demographic differences of sympathetic ophthalmia (SO) in the literature.

Author/year	Total: male (%)/female (%) *P* value	Age: mean ± SD (range, years)	Cause of SO	Race or region	Reference
Chan et al./1995	32: 16 (50)/16 (50) *P* = 1.0000	32.7 ± 23.6 (1–80)	Trauma: 23 (71.9%) Surgery: 9 (28.1%)	NA	[74]

Lin and Zhong/1996	30: 21 (70)/9 (30) *P* = 0.0235*	32.3 (6–66)	Trauma: 24 (80%) Surgery: 6 (20%)	Chinese	[71]

Castiblanco and Adelman/2009	86: 62 (72.1)/24 (27.9) *P* = 0.2655	46 (3–83)	Trauma: 40 (46.5%) Surgery: 38 (44.2%) Trauma + surgery: 8 (9.3%)	NA	[72]

Galor et al./2009	85: 50 (60)/35 (40) *P* = 0.0633	44 (2–91)	Trauma: 53 (62.4%) Surgery: 32 (37.6)	White: 57% African American: 23% Others: 20%	[73]

Al-Halafi et al./2011	60: 34 (56.7)/26 (43.3) *P* = 0.2992	36 ± 20 (4–90)	NA	Saudi Arabia	[8]

*P < 0.05.
